# Knockdown of FOXA2 enhances the osteogenic differentiation of bone marrow-derived mesenchymal stem cells partly via activation of the ERK signalling pathway

**DOI:** 10.1038/s41419-018-0857-6

**Published:** 2018-08-06

**Authors:** Chenyi Ye, Mo Chen, Erman Chen, Weixu Li, Shengdong Wang, Qianhai Ding, Cong Wang, Chenhe Zhou, Lan Tang, Weiduo Hou, Kai Hang, Rongxin He, Zhijun Pan, Wei Zhang

**Affiliations:** 1 Department of Orthopedic Surgery, The Second Affiliated Hospital, School of Medicine, Zhejiang University, No. 88, Jiefang Road, Hangzhou, 310009 China; 2 Orthopedics Research Institute of Zhejiang University, No. 88, Jiefang Road, Hangzhou, 310009 China; 3 Department of Rheumatology, The Second Affiliated Hospital, School of Medicine, Zhejiang University, Hangzhou, China

## Abstract

Forkhead box protein A2 (FOXA2) is a core transcription factor that controls cell differentiation and may have an important role in bone metabolism. However, the role of FOXA2 during osteogenic differentiation of bone marrow-derived mesenchymal stem cells (BMSCs) remains largely unknown. In this study, decreased expression of FOXA2 was observed during osteogenic differentiation of rat BMSCs (rBMSCs). FOXA2 knockdown significantly increased osteoblast-specific gene expression, the number of mineral deposits and alkaline phosphatase activity, whereas FOXA2 overexpression inhibited osteogenesis-specific activities. Moreover, extracellular signal-regulated protein kinase (ERK) signalling was upregulated following knockdown of FOXA2. The enhanced osteogenesis due to FOXA2 knockdown was partially rescued by an ERK inhibitor. Using a rat tibial defect model, a rBMSC sheet containing knocked down FOXA2 significantly improved bone healing. Collectively, these findings indicated that FOXA2 had an essential role in osteogenic differentiation of BMSCs, partly by activation of the ERK signalling pathway.

## Introduction

Fracture delayed union or non-union is one of the most common complications following fixation, which continues to be a main challenge for orthopaedic surgeons. It has been reported that the overall percentage of delayed union or non-union can be as high as 4.4% of all open fractures^1^. Despite significant advances in treatments including autologous bone grafting, artificial bone grafting and commercially available recombinant human bone morphogenetic protein-2, the prognoses for delayed union or non-union are still not optimal^2–5^. Thus, there remains an urgent need to develop new and effective methods to accelerate bone healing.

As a major contributor to bone formation, mesenchymal stem cells (MSCs) have been reported to have a key role during fracture healing and have emerged as the most promising candidate for tissue repair^6–8^. MSCs are regulated by transcription factors and possess self-renewal capabilities, together with the potential to differentiate into a variety of bone formation-related cell types such as osteoblasts and chondrocytes^9–11^. During the fracture-healing process, MSCs are recruited to the fracture area and initiate proliferation and differentiation under the stimulation of the local microenvironment^9,10,12^. The underlying mechanism of osteogenesis, however, remains largely unknown. A better understanding of the osteogenic differentiation of MSCs, especially the involvement of genetic factors, is necessary for their clinical application.

Forkhead box protein A2 (FOXA2), also known as HNF-3β, is a member of the forkhead/winged-helix family of transcription factors (FOXA1, FOXA2 and FOXA3). The members of this family have diverse roles in regulating cell differentiation, development and tissue homoeostasis^13,14^. FOXA2 binds to nucleosomal DNA and is associated with nucleosomal depletion during differentiation^15^. Several studies have reported that FOXA2 closely interacts with several crucial bone metabolism-related genes including *SIRT1*, *SOX9*, *SOX17*, *WNT3* and *WNT7b*, which suggests a potential role of Foxa2 in osteogenesis^16–20^. Among them, *SIRT1* is a repressor of sclerostin and serves as an important regulator of bone mass^21^, whereas *WNT3* and *WNT7b* are required for osteogenesis during biogenesis via Wnt/β-catenin signalling^22,23^. Previous studies have also shown an essential role of *SOX9* and *SOX17* in the proliferation and osteogenesis of stem cells^24,25^. Taken together, there is a potential connection between FOXA2 and bone metabolism, although no related studies have been reported.

In this study, we investigated the effects of FOXA2 on the osteogenic differentiation of bone marrow-derived MSCs (BMSCs). FOXA2 knockdown (KD) enhanced osteogenic differentiation of BMSCs via activation of the extracellular signal-regulated protein kinase (ERK) signalling pathway in vitro, whereas FOXA2 overexpression (OE) inhibited in vitro osteogenic differentiation. Moreover, using a rat tibial osteotomy model, we showed that FOXA2-KD in BMSCs improved bone healing in vivo.

## Results

### Level of FOXA2 was decreased in osteogenic-differentiated rBMSCs compared with undifferentiated BMSCs

**Fig. 1:**
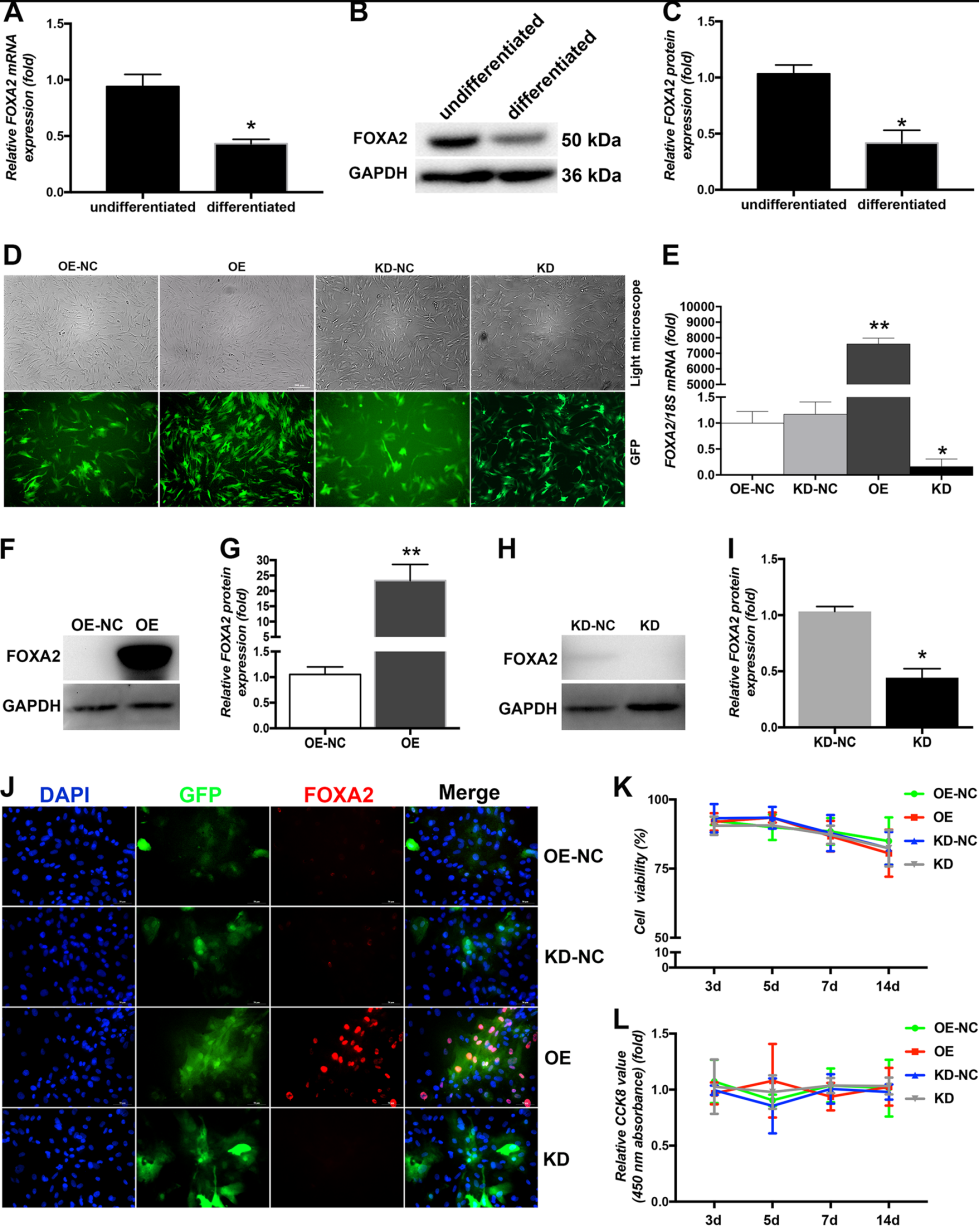
Verification of FOXA2 overexpression and knockdown in BMSCs. **a** FOXA2 mRNA levels were decreased in differentiated BMSCs. **b** FOXA2 protein levels were decreased in differentiated BMSCs. **c** Relative quantitative comparison of western blot analyses mentioned in (**b**). Data are expressed as the mean ± SD. *P < 0.05 vs. undifferentiated BMSCs. **d** rBMSCs after lentiviral transfection and puromycin screening were observed under a normal microscope and a fluorescence microscope. Scale bars, 200 μm. **e** FOXA2 mRNA levels were significantly up- or downregulated relative to the negative-control groups. **f** FOXA2 protein levels were significantly upregulated relative to the negative control group using FOXA2-overexpressing-lentiviral particles. **g** Relative quantitative comparison of western blot analyses mentioned in (**f**). **h** FOXA2 protein levels were significantly downregulated relative to the negative control group using FOXA2-knockdown lentiviral particles. **i** Relative quantitative comparison of western blot analyses mentioned in (**h**). **j** IF analysis confirmed the successful up- or downregulation of FOXA2 protein levels. Scale bars, 50 μm. **k** BMSC proliferation was identified by Trypan blue staining. **l** BMSC proliferation was identified by the CCK-8 assay. Data are expressed as the mean ± SD. *P < 0.05 vs. OE-NC. KD knockdown of FOXA2, KD-NC negative control of FOXA2 knockdown, OE overexpression of FOXA2, OE-NC negative control of FOXA2 overexpression

To determine the expression level of FOXA2 associated with osteogenic differentiation of MSCs, we compared endogenous FOXA2 expression of undifferentiated and osteogenic differentiated rat BMSCs (rBMSCs). Compared with undifferentiated BMSCs, the expression of FOXA2 increased significantly after osteogenic differentiation (Figure 1a‒c).

### Establishment of FOXA2 OE and KD in rBMSCs

To clarify the role of FOXA2 during osteogenic differentiation of BMSCs, endogenous FOXA2 was overexpressed in BMSCs or downregulated by lentiviral particles. Based on its effectiveness, shRNA2 was chosen to downregulate the expression of FOXA2 in subsequent experiments. The lentiviral vectors were efficiently used to overexpress or knock down *FOXA2* in > 80% of third-generation rBMSCs, which was quantified by evaluating the ratio of green fluorescent protein (GFP)-positive cells to the total cell number (Fig. 1d). FOXA2 expression was determined by quantitative PCR (qPCR), immunofluorescence (IF), and western blotting 5 days after infection and screening. FOXA2 mRNA and protein levels were significantly up- or downregulated relative to the negative control groups (Figure 1e‒i), which was also confirmed by IF analysis (Fig. 1j).

### FOXA2 OE and KD did not affect rBMSC proliferation

To determine whether up- or downregulated FOXA2 expression influenced cell viability and proliferation, rBMSC viability was analysed on days 3, 5, 7 and 14 following infection with Trypan blue and Cell Counting Kit-8 (CCK-8) staining. No significant difference in cell viability or proliferation was detected between the FOXA2 OE and normal control (NC) groups. There was also no significant difference in cell viability or proliferation between the FOXA2-KD and KD-NC groups (Fig. 1k, l).

### FOXA2-KD increased the levels of osteospecific genes and proteins, but FOXA2 OE reduced the levels of osteospecific genes and proteins

**Fig. 2:**
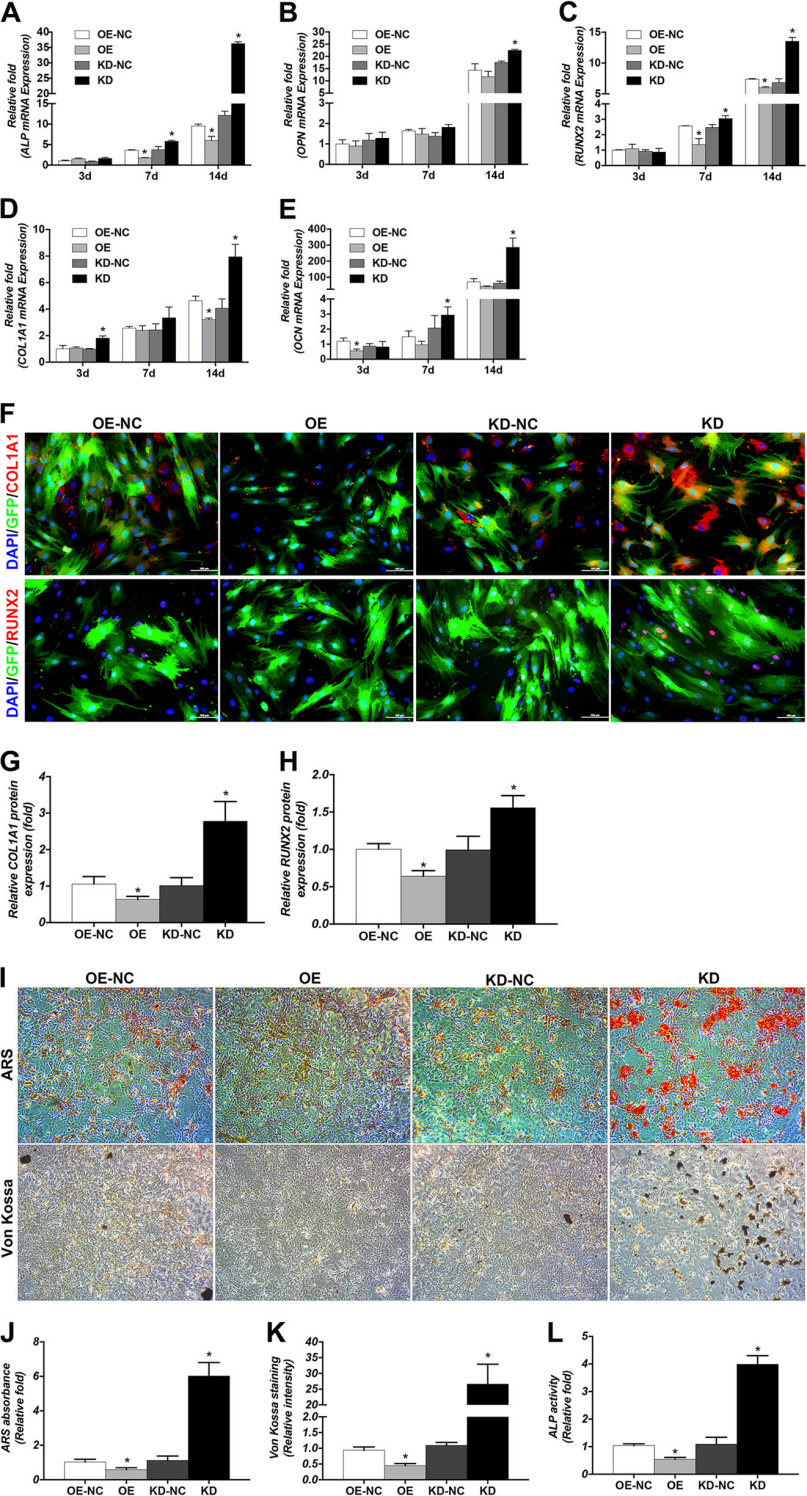
Effects of FOXA2 on osteogenic differentiation of BMSCs. **a**–**e** Relative mRNA expression of osteospecific genes (ALP, OPN, RUNX2, COL1A1 and OCN) on d 3, 7 and 14 of osteogenesis. The mRNA expression levels were normalised to that of 18S ribosomal RNA. **f** Relative expression of osteospecific proteins (RUNX2 and COL1A1) (red) determined by immunofluorescence on day 3 of osteogenesis. Nuclei were counterstained with DAPI (blue). Scale bars, 100 μm. **g** Relative quantitative analysis of COL1A1 immunofluorescence staining. **h** Relative quantitative analysis of RUNX2 immunofluorescence staining. **i** Alizarin red staining on day 10 of osteogenic differentiation. Scale bars, 200 μm. von Kossa staining on day 10 of osteogenic differentiation. Scale bars, 500 μm. **j** Relative quantitative analysis of ARS. **k** Relative quantitative analysis of von Kossa staining. **l** ALP activity detection on day 3 of osteogenic differentiation. Data are expressed as the mean ± SD. KD knockdown of FOXA2, KD-NC negative control of FOXA2 knockdown, OE overexpression of FOXA2, OE-NC negative control of FOXA2 overexpression

To assess the role of FOXA2 in osteogenic differentiation, the levels of osteospecific genes including alkaline phosphatase (*ALP*), osteopontin (*OPN*), *RUNX2*, *COL1A1* and osteocalcin (*OCN*) were detected by qPCR. In addition, the protein levels of the osteospecific markers RUNX2 and COL1A1 were evaluated by IF. The results of qPCR revealed that *ALP*, *OPN*, *RUNX2*, *COL1A1*, and *OCN* mRNA levels were significantly higher in the KD group on days 3, 7 and 14 compared with the KD-NC group (*P* < 0.05). Moreover, lower mRNA levels of *ALP*, *OPN*, *RUNX2*, *COL1A1* and *OCN* were detected in the OE group compared with the OE-NC group (*P* < 0.05) (Figure 2a‒e). We also used IF to confirm the expression of RUNX2 and COL1A1 proteins and determined that their expressions were increased on day 3 in FOXA2-KD BMSCs (Fig. 2f–h).

### FOXA2-KD enhanced calcium deposit formation and ALP activity, whereas FOXA2 OE decreased ALP activity and calcium deposit formation

We evaluated the calcium deposits by Alizarin Red S (ARS) and von Kossa staining. More calcium deposits were present in the KD group than in the KD-NC group on day 10 and less calcium deposits were detected in the OE group than in the OE-NC group (Fig. 2I–K). The activity of ALP, an early marker of osteogenesis, was also examined on day 3 of osteogenic differentiation. Compared with the KD-NC group, increased ALP activity was observed in the KD group (*P* < 0.05), and compared with the OE-NC group, decreased ALP activity was observed in the OE group (*P* < 0.05) (Fig. 2l).

### FOXA2-KD activated the ERK signalling pathway

**Fig. 3:**
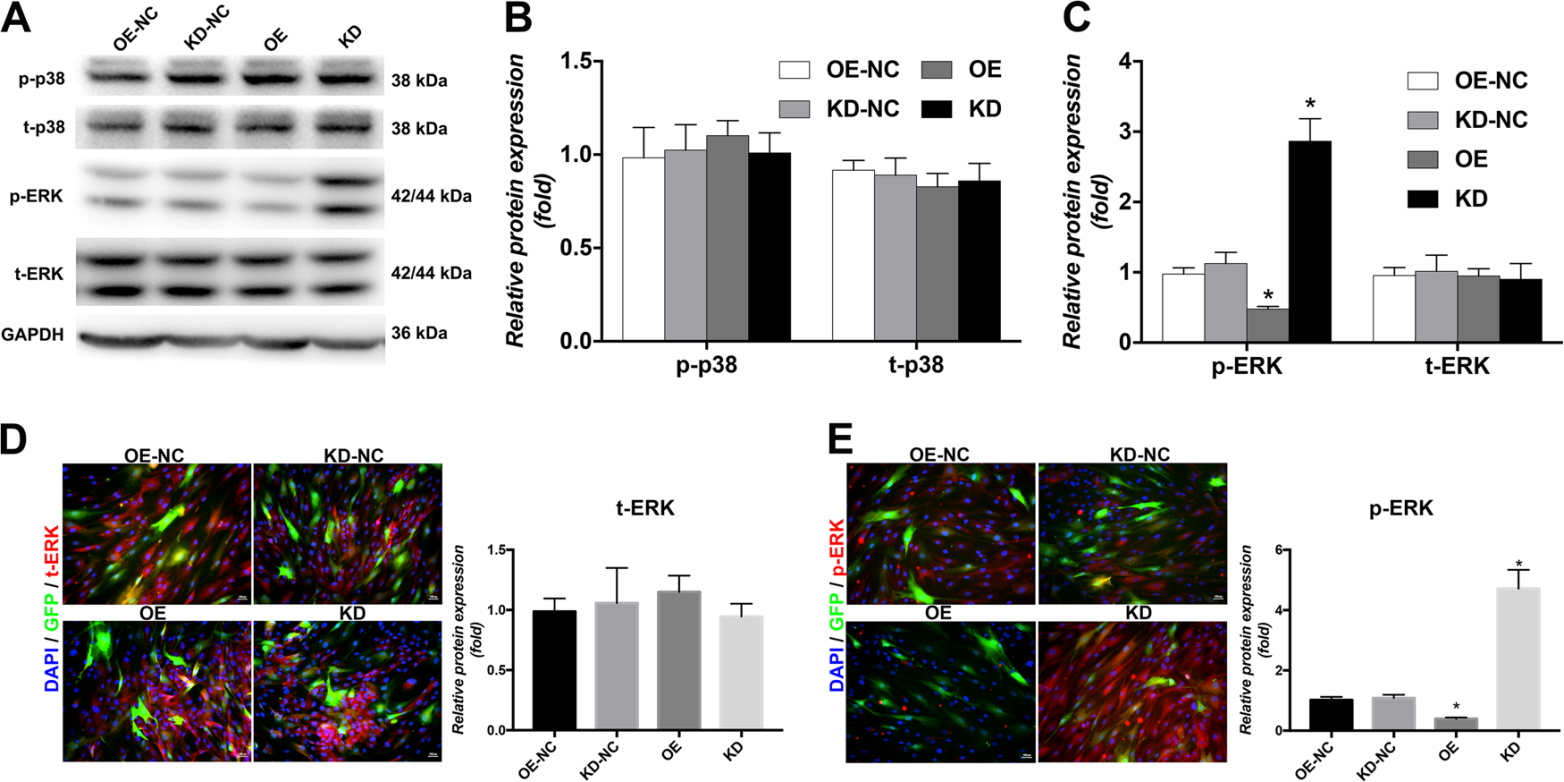
FOXA2 knockdown activated the ERK signalling pathway. **a** Comparison of relevant expression levels of signalling pathway markers by western blot analyses between OE-NC, OE, KD-NC and KD groups. **b** Relative quantitative analysis of western blot analyses for p-p38 and t-p38. **c** Relative quantitative analysis of western blot analyses for t-ERK and p-ERK. **d** Immunofluorescence staining for t-ERK (red). **e** Immunofluorescence staining for p-ERK (red). Nuclei were counterstained with DAPI (blue). Images are magnified × 200. Data are expressed as the mean ± SD. *P < 0.05 vs. OE-NC. KD knockdown of FOXA2, KD-NC negative control of FOXA2 knockdown, OE-NC negative control of FOXA2 overexpression, OE overexpression of FOXA2

To explore the signalling pathways involved in the regulation of BMSC differentiation by FOXA2, one of the most common signalling pathways involved in osteogenesis, the mitogen-activated protein kinase (MAPK) signalling pathway was examined by western blotting. Increased expression of phosphorylated-ERK (p-ERK) was observed in the KD group on day 3 of osteogenic differentiation. Compared with the OE-NC group, the p-ERK level was significantly reduced in the OE group. No significant differences were found in the levels of total ERK (t-ERK), p-p38 and t-p38 among these groups (Figure 3a‒c). Moreover, IF confirmed increased expression of p-ERK accumulation in the KD group and less p-ERK in the OE group compared with the control group. No significant differences in the expression of t-ERK accumulation were observed (Figure 3d‒e).

### Enhanced osteogenic differentiation of BMSCs due to FOXA2-KD was partially reduced by the addition of ERK signalling inhibitors

**Fig. 4:**
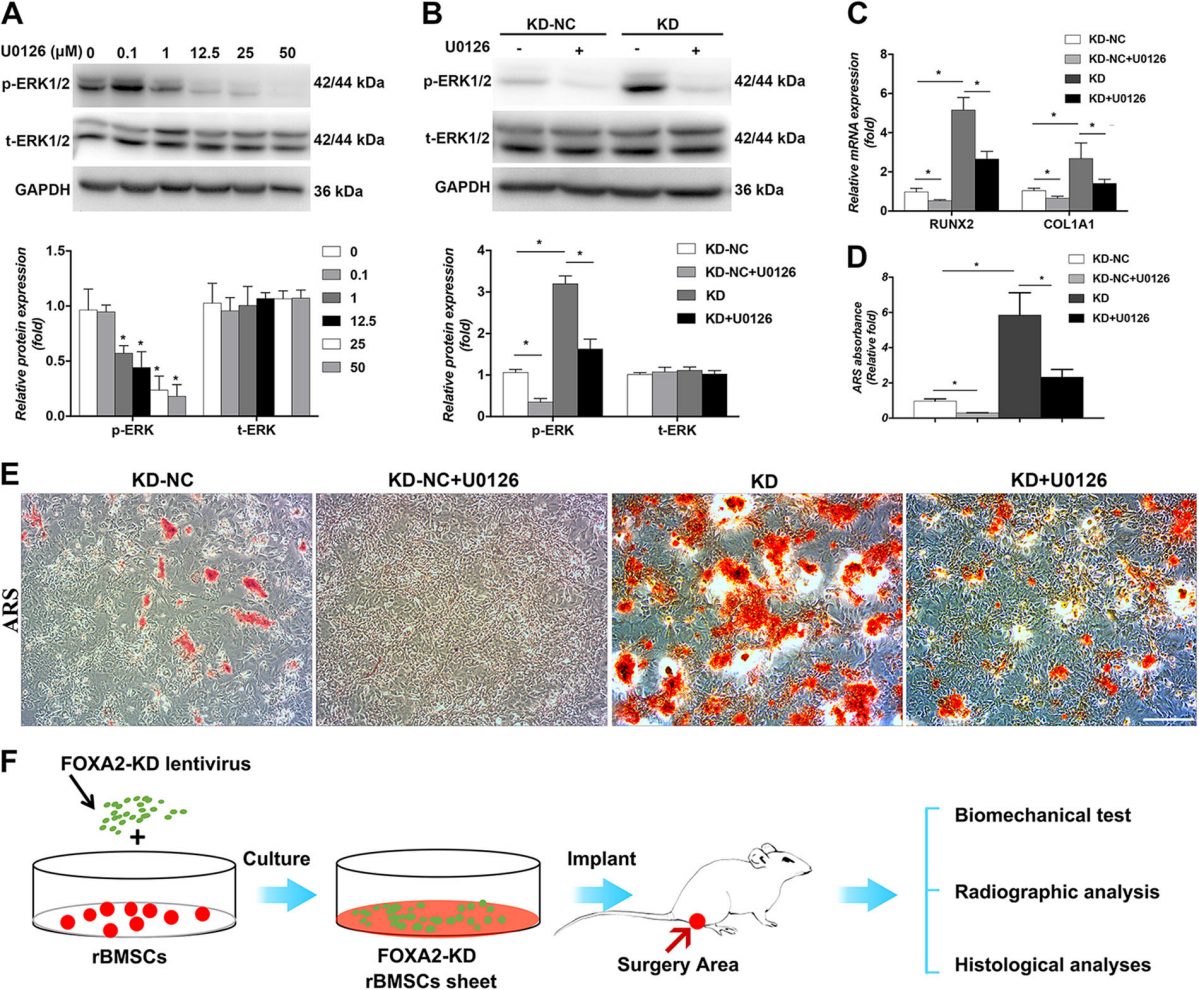
Enhanced osteogenic differentiation of BMSCs due to FOXA2 knockdown was partially reduced by the addition of ERK/MAPK signalling inhibitors. **a** After the addition of U0126 for 24 h, p-ERK was markedly inhibited after treatment with at least 12.5 μM. **b** After the addition of U0126 for 3 days, the level of p-ERK was significantly decreased compared with the level in FOXA2 knockdown rBMSCs without the inhibitor. **c** Inhibition of ERK/MAPK partially reversed the increase of osteospecific genes (RUNX2 and COL1A1). **d** Quatitive analysis of ARS revealed more mineral deposits in FOXA2 knockdown rBMSCs than in the KD + U0126 group. **e** ARS revealed more mineral deposits in FOXA2 knockdown rBMSCs than in the KD + U0126 group. Scale bars, 500 μm. **f** The study design of in vivo evaluation. Data are expressed as the mean ± SD. *P < 0.05. KD knockdown of FOXA2, KD-NC negative control of FOXA2 knockdown

To verify the involvement of the ERK signalling pathway, we evaluated the inhibitory effects of this pathway on osteogenesis in the KD group. The appropriate concentration of U0126 used to efficiently inhibit p-ERK levels was screened by western blotting. After the addition of U0126 for 24 h, both p-ERK and t-ERK levels were examined. The level of p-ERK was significantly inhibited following treatment with at least 12.5 μM (Fig. 4a). Thus, we chose 12.5 μM U0126 for subsequent experiments. Following the addition of U0126 for 3 days, the level of p-ERK was significantly decreased compared with the level in FOXA2 knocked down rBMSCs without the inhibitor (Fig. 4b). Moreover, inhibition of ERK partially reversed the increase in osteogenesis of rBMSCs, as indicated by the expression of osteospecific genes (*RUNX2* and *COL1A1*) (Fig. 4c). In addition, ARS revealed more mineral deposits in FOXA2-KD rBMSCs than in the KD + U0126 group (Fig. 4d, e).

### rBMSC sheets with knocked down FOXA2 accelerated bone fracture healing in a rat tibial defect model

**Fig. 5:**
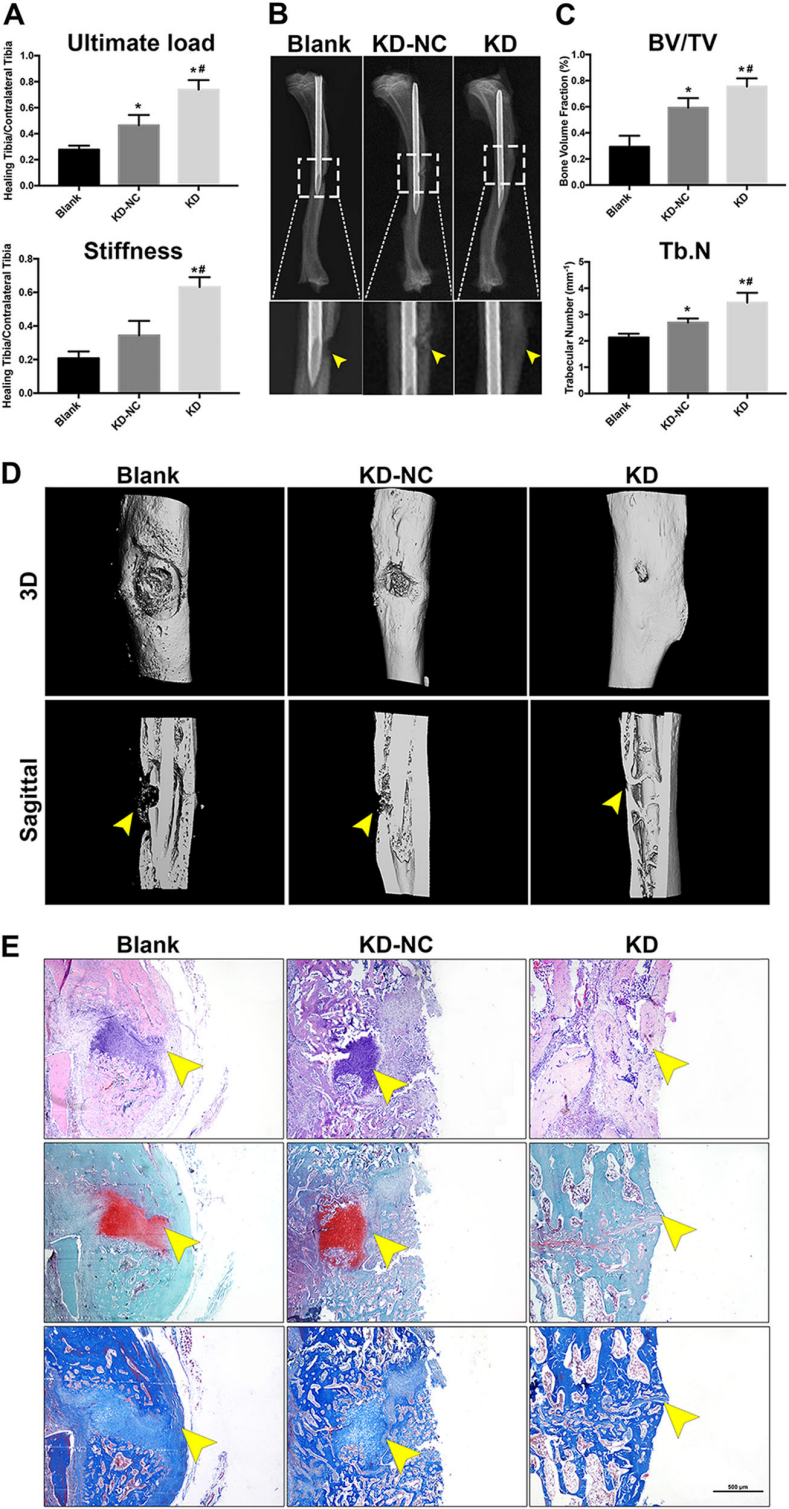
A sheet of rBMSCs with FOXA2 knockdown accelerated bone fracture healing in a rat tibial defect model. **a** The results of biomechanical analysis indicated that treatment with rBMSCs with FOXA2 knockdown markedly increased the ultimate force and stiffness when compared with the blank group and KD-NC group. **b** Radiographic analysis taken at 8 weeks postoperatively. **c** Quatitive μCT analyses of BV/TV and Tb.N. **d** 3D construction images of μCT analyses. **d** Histological evaluation including HE, Safranin O and fast green, and Masson staining of the defect area at 8 weeks after surgery in each group showed that the defects in the blank group were filled with fibrous tissue and a few chondrocytes without bridging bone formation, while a thick callus consisting of newly formed woven bone tissue was observed in the defect area in the KD-NC group. In the FOXA2 knocked down rBMSC group, the defect sites were almost sealed, and remodelling of the callus was more complete. Yellow arrow: the defect area. Scale bars, 500 μm. Data are expressed as the mean ± SD. *P < 0.05 vs. blank group; ^#^*P* < 0.05 vs. KD-NC. KD knockdown of FOXA2, KD-NC negative control of FOXA2 knockdown

To further evaluate the effect of FOXA2-KD in vivo, a sheet of rBMSCs with knocked down FOXA2 was used in a rat tibial defect model. The effect was confirmed by biomechanical testing, and radiographic and histological analysis (Fig. 4f). The results of biomechanical analyses indicated that treatment with rBMSC sheets significantly increased the ultimate force and stiffness when compared with the blank group. Furthermore, a larger ultimate force and greater stiffness were found in the KD group than in the KD-NC group (Fig. 5a). Radiographs taken at 6 weeks postoperatively showed that the cortical defect was clearly present in the blank group. In the KD-NC group, this gap was smaller, and more bridging callus formation was observed at the fracture site compared with the blank group. In the KD group, the gap was obscure (Fig. 5b).

The results of micro-computed tomography (μCT) analyses showed a significant increase in the bone volume fraction (BV/TV) and trabecular number (Tb.N) in KD-NC and KD groups compared with the blank group. The increase was significantly greater in the KD group than the KD-NC group (Fig. 5c). The results of three-dimensional reconstruction of the μCT results showed that the largest defect appeared in the blank group, whereas a smaller defect was observed in the KD-NC group. In the KD group, the defect almost disappeared, and more bridging callus formation was observed, when compared with those of the other two groups (Fig. 5d).

Histological analyses, including hematoxylin and eosin (HE), Safranin O/Fast Green and Masson’s trichrome staining, showed that the defects in the blank group were filled with fibrous tissue and a few chondrocytes; no bridging bone formation at the defect site was observed. In the KD-NC group, a thick callus consisting of newly formed woven bone tissue was observed in the defect area. In the FOXA2 knocked down rBMSC group, the defect sites were almost sealed, and remodelling of the callus was more complete, indicating bone healing of the defect (Fig. 5e).

## Discussion

In the present study we showed that FOXA2, a member of the forkhead/winged-helix family of transcription factors, which has important roles in cell differentiation, development and tissue homoeostasis, acted as an osteogenic differentiation-suppressing gene in MSCs. We found that endogenous expression of FOXA2 was downregulated in rBMSCs during osteogenesis. We therefore used a FOXA2-KD or OE strategy to promote or inhibit osteogenic differentiation of MSCs and found that FOXA2-KD accelerated osteogenesis of rBMSCs via activation of the ERK signalling pathway in vitro. Moreover, a rBMSC sheet with knocked down FOXA2 accelerated bone fracture healing in a rat bone defect model. These findings indicated that FOXA2-KD enhanced osteogenesis of rBMSCs, at least partially by activation of the ERK signalling pathway.

This study emphasised a novel and pivotal role for FOXA2 in regulating the osteogenic differentiation of BMSCs. The Fox protein family consists of 17 subfamilies of transcription factors that display a winged-helix structure in the DNA-binding region. As an important transcription factor, studies of FOXA2 have mainly focused on its regulation of hepatocyte differentiation and pulmonary morphogenesis. A previous study reported that lentivirus-mediated OE of FOXA2 induced rBMSCs into hepatocytes, when tested in vitro^26^. Glucose metabolism is being increasingly accepted as a predominant factor that influences bone ageing and the differentiation potency of MSCs^27^. Wolfrum et al.^28^ reported that FOXA2 is a critical transcription factor in regulating both lipid and glucose metabolism by modulating expression of various genes, including fatty acid synthase, *scd-1*, and glucose 6-phosphatase in the liver. Recently, Ionescu et al.^29^, using mice engineered to lack expression of FoxA2, reported that FOXA2 directly modified collagen X expression and promoted chondrocyte hypertrophy, which further supported the connection between FoxA2 and bone development. Wnt 3 and WNT7b, which are closely related with bone development, have also been found to be directly regulated by FOXA2^18,19^. To some extent, these previous studies further supported our findings that FOXA2 plays an important role in osteogenesis.

Based on previous studies^30,31^, several lineage-specific regulators of osteogenic differentiation have been identified using various approaches such genetic approaches. For examples, regulators that upregulate the expression Wnt3a/Wnt10b^32^, BMP2^33^, Nell-1^34^, Msx2^35^ and SPARC^36^, etc. have been proved to promote the determination of BMSCs into osteoblasts. It is interesting to notice that data involving other protein families such as SFRP2, NURR1, heparin-binding EGF-like growth factor (HB-EGF) and RHEB in the literature reported similar results to the results of this study^37–40^. Jin et al.^37^ reported that SFRP2 enhances the osteogenic differentiation of apical papilla stem cells by antagonising the canonical WNT pathway. Di Benedetto et al.^38^ reported that downregulation of NURR1 strongly prompted the differentiation of dental pulp stem cells towards the osteoblastogenesis process. Using human adipose-derived MSCs, Ashraf et al.^40^ demonstrated that RHEB increased the osteogenesis via the upregulation of Runx2. In addition, microRNAs were also found to be involved in regulating the osteogenic differentiation. Yu et al.^39^ showed that forced expression of HB-EGF or treatment with HB-EGF was capable of reducing osteogenic differentiation of C2C12 cells, whereas upregulation of miR-1192 enhanced Runx2-induced osteogenesis^39^. In summary, multiple regulators of certain cell lineages are involved in osteogenic differentiation. The effects of FOXA2 on osteogenic differentiation found in this study may also be related with the complicated interaction with other protein families or microRNAs, which indicates that molecular switches of these regulators may prove to be ideal “druggable” targets for future clinical intervention aiming at enhancing bone formation.

MAPKs, which consist of ERK, c-jun NH_2_-terminal kinase and p38 MAPK^41^, are important signal transducers in the regulation of osteogenic differentiation of MSCs and bone metabolism^42^. Among them, the ERK signalling pathway, which is activated during osteogenic differentiation, is of vital importance. A majority of the secreted ligands that modulate osteoblast activity appear to act in part through the ERK pathway^43^. In addition, runx2 and osterix are strongly regulated through ERK phosphorylation^44^. Mice with deletions of Erk1 and Erk2 display dramatically reduced bone mineralisation, demonstrating the important role of ERK in osteoblast mineralisation^45^. With regard to the relationship between FOXA2 and ERK signalling, An et al.^46^ reported that valproic acid increased the expression of FOXA2 in MSCs by activating signal transduction of ERK. Our western blotting and IF analyses showed that the ERK signalling pathway was strongly related to FOXA2-KD-promoted osteogenesis. In addition, to confirm our findings, we also examined whether blocking the ERK signalling pathway decreased the effect of FOXA2-KD on rMSC osteogenesis, using U0126. Treatment with U0126 blocked ERK1/2 phosphorylation and significantly decreased runx2, col1a1 activity and mineralised nodule formation in the FOXA2-KD groups. These results indicated that ERK1/2 had a crucial role during FOXA2-KD-induced osteogenesis in rMSCs.

To the best of our knowledge, this is the first study to characterise the effects of FOXA2 on MSC osteogenic differentiation. However, some limitations of our study should be noted. First, although we investigated the role of FOXA2 on osteogenesis, the role of FOXA2 on another important process, osteoclastogenesis, was not studied. Future studies are needed. Second, the molecular mechanism of osteogenesis is complicated; although we indicated that FOXA2 mediated the ERK signalling pathway to regulate the osteogenic differentiation of BMSCs, other signalling pathways may also be involved. The interaction between FOXA2 and the osteogenic differentiation-related genes, also remains to be uncovered in further studies. Third, the nuclear and cytoplasmic localisation of FOXA2, together with its phosphorylation status, were not studied, which may decrease the robustness of the conclusions of this study. Nonetheless, this study used both OE and KD to explore the role of FOXA2, which provided useful insight into the molecular mechanism of bone development and the potential effects of FOXA2 in regulating the osteogenic differentiation of BMSCs. Future studies using genetically engineered mice or large animals are required to further verify our findings.

## Conclusion

FOXA2-KD enhanced osteogenic differentiation of rBMSCs, partly via activation of the ERK signalling pathway.

## Materials and Methods

### Cell culture and reagents

rBMSCs were purchased from Cyagen Biosciences (RASMX-01001, Guangzhou, China), which were cultured in complete rBMSC growth medium (RASMX-90011, Cyagen Biosciences, Inc., Guangzhou, China). Adherent cells were passaged after reaching 80%–90% confluence. Cells from passages 3–9 were used in subsequent experiments. U0126 was purchased from Cell Signalling Technology (Shanghai, China).

### Lentiviral packaging and cell infection

All the lentiviral particles were prepared by Cyagen Biosciences. Three oligonucleotides (5′-GACGCTGAGCGAGATCTAT-3′, 5′-GCGCTTCAAGTGTGAGAAC-3′ and 5′-GCTGCAGACACTTCCTACT-3′), encoding a 19 nt-long short-hairpin RNA (shRNA) against rat FOXA2, were designed and the most effective one among them was chosen for the following experiments. A scrambled shRNA sequence (5′-TTCTCCGAACGTGTCACGT-3′), exhibiting no homology to the rat sequence database, was used as a negative control [FOXA2-KD control group (KD-NC)]. The oligonucleotides were phosphorylated, annealed and cloned into the pLVX-shRNA vector. The resulting vectors, designated pLVX–rat-shFOXA2 and pLVX–rat-shFOXA2–Control, were subsequently verified by sequencing. Lentivirus overexpressing FOXA2 (lenti-FOXA2) particles and lentiviral GFP particles (lenti-control) were prepared by Cyagen Biosciences. The lentiviral GFP particles were used as control group [FOXA2 OE control group (OE-NC)] in this study. For retroviral OE of FOXA2, the full-length cDNA of human FOXA2 (NM_012743.1) was obtained by PCR. Next, FOXA2 cDNA was subcloned into the BamHI and XhoI sites of the CMV-MCSEGFP-IRES retroviral vector (Cyagen Biosciences).

For infections, rBMSCs were incubated with lentiviral particles and polybrene (5 μg/mL) in growth medium. After about 24 h, the infection medium was discarded. After 3 days, the cells were screened using puromycin (4 μg/mL) and then passaged for use in subsequent experiments. Transduction efficiency was monitored by GFP fluorescence. The expression of FOXA2 was quantified by quantitative real-time PCR and western blot analyses.

### Trypan blue staining

After infection of lentiviral particles, rBMSCs were collected on d 3, 5, 7 and 14. Cell viability was determined using trypan blue staining by an Automated Cell Counter (Countess; Thermo Fisher Scientific, Waltham, MA, USA) as previously described^47^.

### Cell Counting Kit-8

To assess the effect of FOXA2 OE and KD on the proliferation of rBMSCs, the cells were seeded into a 96-well plate (5000/well) and allowed to adhere for 24 h. Then the medium was removed and the cells were treated with 10% CCK-8 (Dojindo, Kumamoto, Japan) in 150 μl L-Dulbecco’s modified Eagle’s medium (DMEM) without fetal bovine srum (FBS) for 3 h at 37 °C. Absorbance at 450 nm was measured using a microplate reader (ELX808; BioTek, Winooski, VT, USA).

### Osteogenic differentiation protocol

For the osteogenic differentiation, rBMSCs were first cultured in growth medium (RASMX-90011, Cyagen Biosciences, Inc.) in 6- or 12-well cell culture plates (Corning, Shanghai, China), at a density of 3 × 10^4^/cm^2^ and incubated for 48 h at 37 °C under 5% CO_2_. The rBMSCs were subsequently cultured in osteogenic induction medium (low-sugar DMEM; 10% FBS (1495527; Gibco, Waltham, MA, USA), 100 nM dexamethasone, 100 IU/ml penicillin/streptomycin, 10 mM β-glycerophosphate and 0.2 mM ascorbic acid), which were then maintained by the addition of fresh osteogenic induction medium every 2–3 days.

### Measurement of ALP activity and ALP staining

For the measurement of ALP activity, osteogenic differentiated cells were lysed in radioimmunoprecipitation assay (RIPA, Beyotime, Shanghai, China) lysis buffer consisting of 20 mM Tris–HCl (pH 7.5), 150 mM NaCl and 1% Triton X-100. Then ALP activity was determined using an ALP Activity Assay (Beyotime) according to the manufacturer’s instructions. Briefly, 10 μl lysate was incubated with 90 μl fresh solution containing p-nitrophenyl phosphate substrate at 37 °C for 30 min. Then, 100 μl 0.5 N NaOH was used to stop the reaction. The absorbance was measured at 405/650 nm on a microplate reader (ELX808; BioTek). For ALP staining, cells were fixed with 4% paraformaldehyde (Sigma, Shanghai, China) for 15 min at room temperature and washed three times with phosphate-buffered saline (PBS). Cells were then stained using a BCIP/NBT ALP Color Development Kit (Beyotime, Shanghai, China).

### ARS staining

For the measurement of mineral deposition after the induction of osteogenic differentiation, ARS was used. Cells were fixed in 4% paraformaldehyde (Sigma) for 15 min at room temperature and then washed three times with PBS. Then, 1% solution of ARS kit (Cyagen Biosciences) was added and incubated for 30 min at room temperature, followed by rinsing with PBS. The solution was collected and the absorbance at 560 nm of 200 μl solution of stained cells in 96-well plates was read using a microplate reader (ELX808; BioTek). The readings were normalised to the total protein concentrations.

### Von Kossa staining

For von Kossa staining, osteogenic differentiated cells were fixed with 4% paraformaldehyde (Sigma) for 15 min at room temperature and washed three times with PBS. Next, cells were incubated with a 5% silver nitrate solution and were exposed to UV radiation for 30 min. After that, 5% sodium thiosulfate was added for 5 min, to remove nonspecific staining.

### Immunofluorescence

Cells were cultured in a 12-well plate, and FOXA2, RUNX2, COL1A1, t-ERK and p-ERK were detected using a fluorescence microscope (EU5888; Leica, Wetzlar, Germany). Briefly, cells were fixed in 4% paraformaldehyde (Sigma) for 15 min at room temperature, permeabilized and blocked for 30 min in 0.05% Triton X-100 and 5% bovine serum albumin (BSA). Fixed cells were washed three times with PBS and incubated at 4 °C overnight with anti-FOXA2 (1:400; Cell Signalling Technology), RUNX2 (1:1600; Cell Signalling Technology), COL1A1 (1:500; Abcam, Shanghai, China), t-ERK (1:800; Cell Signalling Technology) or p-ERK (1:200; Cell Signalling Technology). Cells were incubated with a fluorescence-conjugated secondary antibody (Beyotime) at room temperature for 2 h and nuclei were stained with 4′,6-diamidino-2-phenylindole (KeyGen Biotech, Nanjing, China) for 4 min. Samples were then observed and photographed under a fluorescence microscope (Leica).

### RNA isolation and qPCR

Total cellular RNA was isolated using RNAiso reagent (Takara, Dalian, China) and quantified by measuring the absorbance at 260 nm (NanoDrop 2000; Thermo Fisher Scientific). Total RNA ( ≤ 1000 ng) was then reverse-transcribed into cDNA in a reaction volume of 10 μl using a Double-Strand cDNA Synthesis Kit (Takara). All gene transcripts were quantified by qPCR using the Power SYBR^®^ Green PCR Master Mix (Takara) on the ABI StepOnePlus System (Applied Biosystems, Warrington, UK). 18 S was used as a housekeeping gene. The mRNAs of the target genes and the housekeeping gene were quantified in separate tubes. All primers used in this study were synthesised by Sangon Biotech (Shanghai, China), the primer sequences of which were shown in Table 1. The cycle conditions of qPCR were set as follows: 95 °C for 30 s and then 42 cycles of 95 °C for 5 s and 60 °C for 30 s. The 2^−△△Ct^ method was used to calculate the relative expression levels of target genes.

**Table 1:** Sequences of primers for quantitative real-time PCR

Gene	Reverse (5′–3′)	Reverse (3′–5′)
FOXA2	CACGGCTCCCAGCATACTTT	CACGGCTCCCAGCATACTTT
ALP	GCCGGCCCAAGAGAGAA	CCGATGGGACCGTGGTT
RUNX2	CAGCAGAGGCATTTCGTAGCT	CATCGGTGGTACTAAC
COL1A1	CTGGATCATATTGCACA	GAGCTGCCCTGCACTGGGTG
OCN	TGGCCCCAGACCTCTTCCCG	TGGCCCCAGACCTCTTCCCG
OPN	CAGGCTGGCTTTGGAACT	CAGGCTGGCTTTGGAACT
18 S	TTGACGGAAGGGCACCA	TTGACGGAAGGGCACCA

### Western blot analysis

Protein extracts from cells were prepared in RIPA lysis buffer supplemented with a proteasome inhibitor (Beyotime, Haimen, China). Total proteins were separated by 10% SDS-polyacrylamide gel electrophoresis and then transferred to a polyvinylidene difluoride membrane (Millipore, Shanghai, China). After blocking in 5% BSA for 1 h at room temperature, the membranes were incubated overnight at 4 °C with antibodies specific to GAPDH (1:2000, Cell Signalling Technology), FOXA2 (1:1000; Cell Signalling Technology), RUNX2 (1:1000; Cell Signalling), COL1A1 (1:1000; Abcam), t-ERK (1:1000; Cell Signalling Technology) or p-ERK (1:2000; Cell Signalling Technology). Horseradish peroxidase-conjugated goat anti-rabbit IgG (1:5000, Boster Biologic Technology, Wuhan, China) was used as a secondary antibody for 2 h at room temperature. The immunoreactive bands were visualised using an enhanced chemiluminescent detection reagent (Millipore). Signal intensity was quantified using a Bio-Rad XRS chemiluminescence detection system (Bio-Rad, Hercules, CA, USA).

### Cell sheet preparation

The protocol of cell sheet preparation was according to our previously reported studies^48,49^. Briefly, confluent cells (1 × 10^5^/cm^2^) in flasks were cultured in MSC growth medium with the addition of vitamin C (20 µg/mL) for 2 weeks to form a sheet of rBMSCs. Cells were then rinsed twice with PBS, and then detached intact from the substratum as cell sheets using a scraper.

### In vivo evaluation in animals

All Sprague–Dawley (SD) rats were supplied by the Academy of Medical Sciences of Zhejiang Province. The protocol of all animal experiments was approved by the Institutional Animal Care and Use Committee of the Second Affiliated Hospital, School of Medicine, Zhejiang University. All animal experiments were performed in accordance with the Animal Care and Use Committee guidelines of Zhejiang province, strictly following the guidelines for the care and use of laboratory animals. The animals had free access to food and water and were kept in a pathogen-free animal room.

In total, 30 male SD rats weighing (8-week-old, weighing 250–300 g) were used to establish a rat tibial defect model. The rats were divided randomly and evenly into three groups: a blank group, an KD-NC (negative-control group of rBMSCs with FOXA2-KD) group and an KD (rBMSCs with FOXA2-KD) group (*n* = 10 per group). All surgical procedures were performed by two experienced orthopaedic surgeons. The tibial defect model was established as reported previously^50,51^. Briefly, rats were anaesthetised intraperitoneally with 0.3% pentobarbital sodium (Sigma) at 30 mg/kg body weight. After anaesthesia, an incision was made below the knee, which was followed by an intramedullary needle (1.2 mm-diameter stainless steel syringe needle) being inserted inside the medullary canal of the tibia for fixation^52^. A 1.5 mm-diameter tibial defect was made in all SD rats approximately 1 cm from the proximal tibial growth plate by a hollow drill^51–53^. The same leg was used for each group. As is introduced above, cell sheets with FOXA2-KD and the negative controls were prepared with confluent cells (1 × 10^5^/cm^2^) in flasks for 2 weeks before use. The details of cell sheet implantation were introduced in our previous studies^48,49,54^. In brief, in the blank group (*n* = 10), nothing was grafted onto the tibial defect site; in the KD-NC group, a sheet of KD-NC rBMSCs were used to fill the defects and wrap around the defect areas; in the KD group, a sheet of rBMSCs with FOXA2-KD was implanted into the defects and wrap around the defect areas (Fig. 4f).

All of the rats were sacrificed in a CO_2_ chamber at 6 weeks postoperatively. Specimens of the experimental and the contralateral intact tibias were collected for biomechanical testing, radiographic analysis, and histological analysis. For biomechanical testing, specimens (*n* = 5 each group) were immediately frozen at − 80 °C and thawed overnight at 4 °C for testing. For radiographic and histological analysis, specimens (*n* = 5 each group) were fixed in 4% paraformaldehyde (Sigma) for 48 h at room temperature and kept in PBS.

### Biomechanical testing

To evaluate the function of the experimental tibia, a three-point bending biomechanical test was performed as previously described^48,55^. Both the experimental and the contralateral intact tibias were tested compared using a Zwick/Roell 2.5 material testing system (Zwick, Ulm, Germany). All the tibia samples were kept moist with normal saline during biomechanical testing. A preloaded of 2 N at a speed of 0.2 mm/s was applied to adapt for 10 s. Thereafter, tibia samples from rats were tested using a vertical pushing load until failure at a constant speed of 1.0 mm/s. The time, force, and displacement until failure were recorded. The ultimate load, stiffness and absorbed energy were determined and calculated based on the force-displacement curve,

### Radiographic analysis

To evaluate callus formation and bridging bone formation at the fracture sites 6 weeks postoperatively, radiographs (*n* = 5 for each group) were taken using a dual-track molybdenum/rhodium + Mo target mammography machine (22 KV, 250  mAS; GE, Fairfield, CT, USA). For the μCT evaluation, tibia samples were scanned using a μCT-100 imaging system (Scanco Medical, Brüttisellen, Switzerland) with X-ray energy settings of 70 kVp, 1024 reconstruction matrix, 14.8 μm slice thickness with an exposure time of 300 ms. The BV/TV and Tb.N were calculated by three-dimensional standard microstructural analysis^48,56^.

### Histological evaluation

After μCT scanning, the specimens (*n* = 5 for each group) were decalcified in 10% ethylene diaminetetra acetic acid (EDTA, Sigma) with 0.1 M PBS for more than 60 days, with a solution change once a week. Thereafter, the specimens were embedded in paraffin using standard procedures. Serial sections (3 µm thickness) were cut and mounted onto polylysine-coated glass slides, and deparaffinized. Sections were stained with HE, Safranin O/Fast Green and Masson’s trichrome staining were performed separately consecutive tissue sections in accordance with our previous studies^48,52^. Images were obtained on a traditional light microscopy (Leica DM4000B; Leica, Solms, Germany).

### Statistical analysis

Statistical analysis was performed using SPSS 19.0 software (IBM, Armonk, NY, USA). All experiments were carried out at least in triplicate, and the data are presented as means ± SD. To calculate the significant differences, a two-tailed Student’s *t*-test was used when comparing two groups, one-way ANOVA followed by Bonferroni’s post-hoc test was used when comparing more than two groups. A value of *P* ≤ 0.05 was considered to indicate statistical significance.
